# “Toll-free” pathways for production of type I interferons

**DOI:** 10.3934/Allergy.2017.3.143

**Published:** 2017-11-06

**Authors:** Ling Wang, Shunbin Ning

**Affiliations:** 1 Department of Internal Medicine, Quillen College of Medicine, East Tennessee State University, Johnson City, TN 37614, USA; 2 Center of Excellence for Inflammation, Infectious Diseases and Immunity, Quillen College of Medicine, East Tennessee State University, Johnson City, TN 37614, USA

**Keywords:** PRR, cGAS, IFN-I, innate immunity

## Abstract

Pathogen-associated molecular patterns (PAMPs) and damage-associated molecular patterns (DAMPs) are recognized by different cellular pathogen recognition receptors (PRRs), which are expressed on cell membrane or in the cytoplasm of cells of the innate immune system. Nucleic acids derived from pathogens or from certain cellular conditions represent a large category of PAMPs/DAMPs that trigger production of type I interferons (IFN-I) in addition to pro-inflammatory cytokines, by specifically binding to intracellular Toll-like receptors or cytosolic receptors. These cytosolic receptors, which are not related to TLRs and we call them “Toll-free” receptors, include the RNA-sensing RIG-I like receptors (RLRs), the DNA-sensing HIN200 family, and cGAS, amongst others. Viruses have evolved myriad strategies to evoke both host cellular and viral factors to evade IFN-I-mediated innate immune responses, to facilitate their infection, replication, and establishment of latency. This review outlines these “Toll-free” innate immune pathways and recent updates on their regulation, with focus on cellular and viral factors with enzyme activities.

## 1. Introduction

Pathogen-associated molecular patterns (PAMPs) are usually derived from invading pathogens, and initiate rigorous innate immune responses after recognized by host germline-encoded pathogen recognition receptors (PRRs), which are expressed on cell membrane or in the cytoplasm of cells in the innate immune system. PRRs include the well-known transmembrane Toll-like receptors (TLRs), and an increasing pool of Toll-unrelated receptors (so we call them “Toll-free” receptors hereafter) that include retinoic acid-inducible gene I (RIG-I)-like receptors (RLRs), Caterpiller-like receptors (CLRs), the HIN200/PYHIN family of nuclear antigens, cGMP-AMP synthase (cGAS), DNA-dependent activator of IRFs (DAI) [1], DDX9, DDX36 [2], DDX41 [3,4], RNA polymerase III [5], Ku70 [6], MRE11 [7], Sox2 [8], LRRFIP1 [9], ISG56/IFIT1 [10] and OASs [11], amongst others [12–15]. Intracellular PRRs, including endosomal TLRs, are able to recognize nucleic acids to trigger signal cascades for the activation of NFκB and specific interferon regulatory factors (IRFs) leading to production of type I interferons (IFN-I) [15]. Among these “Toll-free” receptors, RLRs and cGAS play non-redundant roles in the recognition of cytosolic RNAs and DNAs respectively, and they govern intracellular IFN-I-mediated innate immune responses. The involvement of other cytosolic “Toll-free” sensing pathways in IFN-I-mediated innate immunity is controversial due to the lack of solid evidence of genetic studies [16]. Importantly, recent studies have shown that the IFN-I signaling pathway plays a dual role in chronic viral infections. At the early stage of infection, it has a potent antiviral activity; however, at late stages, a low level of prolonged IFN-I response facilitates the establishment and maintenance of persistent infection [17–22].

Except PAMPs, host damage-associated molecular patterns (DAMPs), such as self-nuclei acids, heat-shock proteins and HMGB1, are also recognized by PRRs [13]. Self-DNA can be accumulated in the cytoplasm in mammalian cells under stress and specific physiological conditions, including but not limited to apoptosis, DNA damage, and spontaneous aging/senescence. [14,23,24]. Reactive oxygen species (ROS) produced from damaged mitochondria are one of the major cause of DNA damage, especially those with double-strand breaks, apart from other servere effects such as cell-cycle arrest, senescence and cell death [25–29]. Accumulation of damaged DNA fragments is able to activate cGAS- or RIG-I-mediated aberrant production of persistent IFN-I and AIM2-mediated inflammatory responses, causing autoimmune diseases and cancerogenesis [16,30–35]. Persistent IFN-I production also triggers chronic immune activation, and ultimately results in immune exhaustion and senescence, in the setting of persistent infection by viruses such as HIV and HCV [19,36,37].

Furthermore, there is substantial evidence showing that prolonged IFN-I signaling has important roles in regulating T cell responses in both direct and indirect manners, either promoting or inhibiting T cell activation, proliferation, differentiation and survival, and thus serves as a bridge that links innate and adaptive immune responses [19,20,21,38,39,40]. For example, CD4^+^ T cells derived from HIV^+^ subjects display an anergic phenotype, and a recent report has shown that engagement of TLR7 in HIV-infected CD4^+^ T cells induces anergy/unresponsiveness, accounting for the impaired T cell function by chronic HIV infection [41].

In essence, IFN-I production has to be finely tuned to ensure appropriate mounting of antiviral and anti-tumor immunity and to maintain cellular homeostasis. We outline the “Toll-free” pathways triggering IFN-I production in this review, highlighting cGAS, HIN200, and RLRs, and recent updates on their regulation by cellular and viral factors with enzymatic activities.

## 2. “Toll-free” Nucleic Acid-Sensing Innate Immune Pathways

### 2.1. cGAS-STING cytosolic dsDNA-sensing pathway

**Figure 1:**
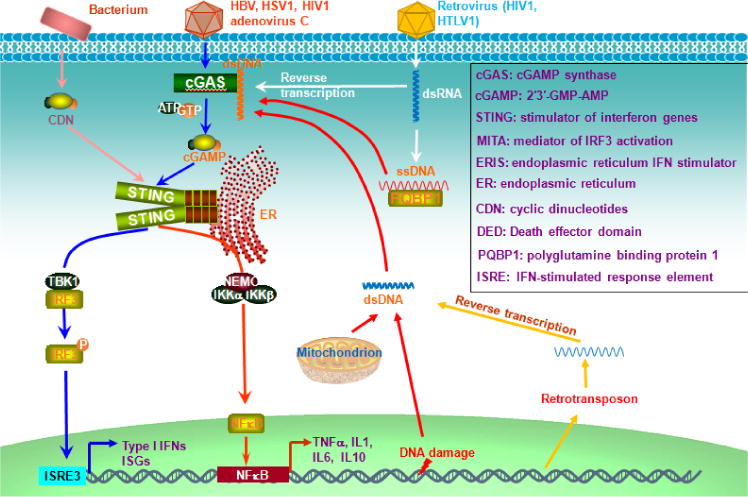
cGAS-STING pathway senses cytosolic dsDNA. Cytosolic dsDNA derived from invading DNA viruses or retroviruses, or from self-genome under stress conditions (such as damaged mitochondrial DNA) binds to cGAS, leading the synthesis of cGAMP from ATP and GTP. cGAMP binds to STING dimers, resulting in STING phosphorylation and activation, further triggering downstream pathways for production of IFN-I and proinflammatory cytokines.

2cGMP-AMP synthase (cGAS) synthesizes 2′3′-GMP-AMP (cGAMP) from ATP and GTP after binding to double-stranded DNA (dsDNA), and is the primary indispensable cytosolic sensor for both exogenous and endogenous dsDNA molecules, which, with the sizes of as short as 20 bp, are recognizable by high concentrations of cGAS or, with long sizes with bounded protein, recognizable by lower concentrations of cGAS [42]. In conjunct with ER-anchored IFN-inducible stimulator of interferon genes (STING, also called MITA, ERIS, MYPR) that functions as an adaptor to bind to cGAMP, cGAS-STING-mediated pathway plays a key role in innate immune responses against a large spectrum of DNA viruses (HSV1, vaccinia virus, adenovirus, KSHV, etc.), retroviruses (HIV1, HIV2, etc.) that produce pro-integrating DNA molecules, as well as bacteria (*Mycobacteria, Legionella, Listeria*, etc.) (Figure 1). In addition to viral infection, chemotherapeutic agents such as cisplatin, etoposide, and chitosan, induce oxidative DNA damage that promotes self-DNA leakage from the nucleus and mitochondrion, and therefore trigger cGAS-STING-mediated antitumor immune response (Figure 1) [28]. dsDNA molecules derived from retrotransposons such as short interspersed nuclear elements (SINEs) and Alu elements, can also function as ligands for cGAS. Approximately half of the mammalian genomes consist of retrotransposons [43].

Binding of cGAMP to two STING protomers results in translocation of STING from the ER compartment to perinuclear autophagy-like vesicles, where TBK1 is autophosphorylated and recruited to STING, allowing STING to be phosphorylated at Ser366, which is in an evolutionary conserved serine cluster shared with MAVS, TRIF, and IRF3 so that they all are also activated through similar phosphorylation-dependent mechanism [44]. Phosphorylated STING further recruits IRF3, resulting in its phosphorylation and activation by TBK1. cGAMP can be transferred to adjacent non-infected cells to spread antiviral activity, by diffusion or by being packed into viral particles, and therefore cGAS-STING can mount unique regional immune response [28].

STING itself is a receptor for bacterial cyclic dinucleotides (CDN) and their binding activates IFN-I responses in mice but not in humans (Figure 1). Binding of CDN to STING dimers causes the relief of STING autoinhibition, which allows STING to be ubiquitinated with K27 chains by the ER-anchored E3 ligases AMFR, further leading to the recruitment of TBK1 for IRF3 activation [45].

### 2.2. HIN200-mediated cytosolic dsDNA-sensing pathways

**Figure 2:**
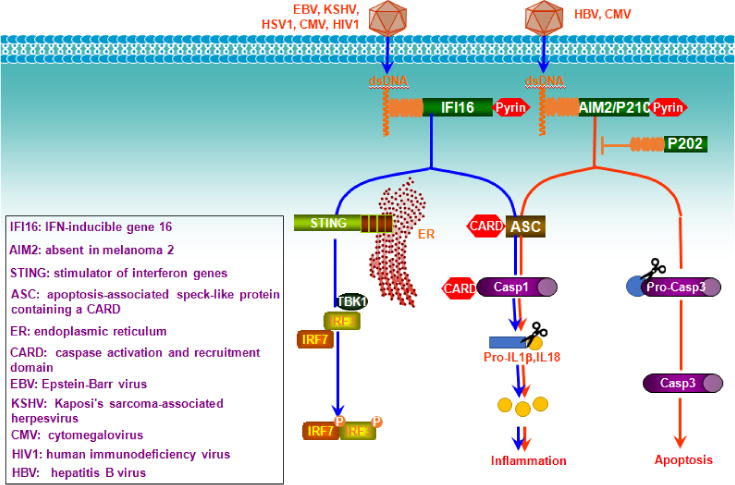
HIN200-mediated cytosolic dsDNA-sensing pathways. The dsDNA sensors AIM2 and IFI16 in the HIN200 family recognize long viral genome DNA in the cytoplasm, triggering caspase 3-mediated apoptosis and caspase 1-mediated inflammation. Recognition of dsDNA by IFI16 also triggers STING-mediated IRF3/7 activation and IFN-I production. While AIM2 activates caspases to trigger apoptosis and inflammation, mouse P202 inhibits AIM2-mediated pathways.

Except cGAS, two other important receptors for cytosolic dsDNA, AIM2 and IFI16, belong to the HIN200/PYHIN family that includes 4 members in humans: AIM2 (PYHIN4), IFI16 (PYHIN2), IFIX (PYHIN1), and MNDA (PYHIN3). The members are hematopoietic interferon-inducible nuclear antigens with 200 amino acid repeats and an N-terminal PYHIN (Pyrin and HIN) domain (except p202 in mice). Upon binding to DNA, both AIM2 and IFI16 trigger signal transduction to activate caspase 1 inflammasome leading to cleavage of pro-IL1β and pro-IL18 into mature bioactive IL1β and IL18 via the CARD-containing adaptor ASC (CARD5) [46]. IFI16 preferentially recognizes longer naked DNA (>150 bp), and also triggers IFN-I responses via STING, in addition to its ability to induce caspase 1 inflammasome. AIM2 also triggers signal to caspase3-dependent apoptosis (Figure 2). Like cGAS, IFI16 and AIM2 bind to DNA independent of the DNA sequence [46].

Infection of a DNA virus activates both cGAS and IFI16 in the cytoplasm. However, recent studies support that DNA viral genome initiates IFI16-mediated immune response in the nucleus, where episomal genomes of almost all DNA viruses residue and replicate [47]. At the same time, viral infection (such as herpesviruses) imposes cellular stress that triggers mitochondrial DNA damage and release to activate cGAS-STING pathway [48]. Of special interest, Epstein-Barr Virus (EBV) latent infection, which is associated with various malignancies, constitutively induces IFI16-ASC-caspase 1 inflammasome that may represent a chronic inflammatory microenvironment for EBV-mediated oncogenesis [49].

Single-stranded DNA (ssDNA) derived from retroviral genome reverse transcription or from DNA replication (named Y-form DNA) potently stimulates IFN-I responses depending on cGAS or IFI16; whereas the RNA:DNA intermediate derived from reverse transcription prior to host genomic integration is detected by TLR9 and cGAS [50].

The other two HIN200 family members, IFIX and MNDA, are less studied but existing evidence has shown that they are involved in regulation of IFN signaling in cancers. IFIX promotes ubiquitination-mediated degradation of MDM2, leading to p53 stabilization, and stimulates maspin expression by promoting ubiquitination-mediated degradation of HDAC1, leading to impaired invasion activity of cancer cells.

### 2.3. RLR cytosolic RNA-sensing pathways

**Figure 3:**
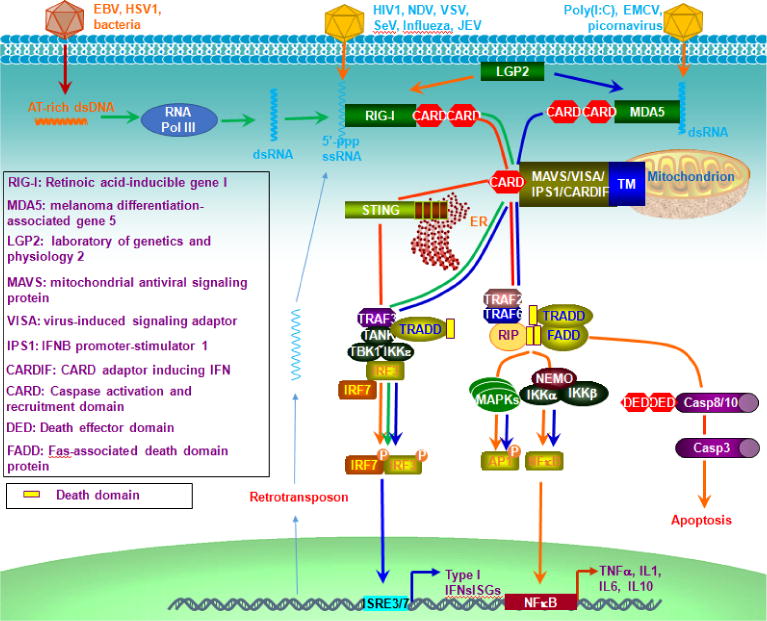
RLR-mediated RNA sensing pathways. RIG-I and MDA5 recognize different patterns of cytosolic RNA fragments derived from viruses, bacteria, or the host cell. STING interacts with RIG-I and MAVS in a complex that is stabilized upon RNA virus infection, facilitating the antiviral response. In addition, AT-rich dsDNA derived from bacteria or DNA viruses can be transcribed by RNA polymerase III into 5′-ppp dsRNA, which activates RIG-I-MAVS-TBK1-IRF3 pathway for IFN-I production.

RLRs include RIG-I (DDX58), MDA5 (IFIH1), and LGP2, all of which contain a central DEAD box helicase/ATPase domain and a C-terminal regulatory domain (CTD); the latter (CTD) is essential for RNA binding. RIG-I and MDA5 each contains 2 CARD domains, and both signal through the mitochondrial CARD-containing antiviral signaling adaptor MAVS (also called VISA, IPS1, or CARDIF) that is located on mitochondrial membrane. RIG-I recognizes dsRNA synthesized from AT-rich dsDNA derived from DNA viruses (EBV, HSV1) and bacteria [5], and recognizes 5′-ppp ssRNA derived from the RNA viruses NDV, VSV, Sendai virus, influenza A virus and JEV; while MDA5 recognizes >300 bp long dsRNA such as poly (I:C) and RNA from EMCV and paramyxovirus (Figure 3) [51]. Solid evidence have shown that STING is also involved in RIG-I signaling, and interacts with MAVS in a complex that is stabilized in response to RNA virus infection, potentiating RIG-I-mediated antiviral responses (Figure 3) [51]. In the absence of ligand challenges, RIG-I and MDA5 are inactive due to phosphorylation at specific Thr and Ser residues (Ser8 and Thr170 of RIG-I, and Ser88 of MDA5), which is mediated by casein kinase II (CKII) and protein kinase C (PKC)-α/β respectively for RIG-I and by RIOK3 for MDA5. Activation of RIG-I and MDA5 requires their dephosphorylation by PP1α/γ, ubiquitination, and ATP-dependent conformational changes leading to their multimerization upon binding to RNA. The other member, LGP2, can recognize RNA but lacks CARD domains, and results from knockout mice have shown that LGP2 is in fact a positive regulator of RIG-I/MDA5 signaling [52]. Different from TLRs that are restrictedly expressed in immune cells, epithelial cells and synovial fibroblasts, RLRs are expressed in most cell types.

### 2.4. Other “Toll-free” cytosolic innate sensing pathways for IFN-I production

**Figure 4:**
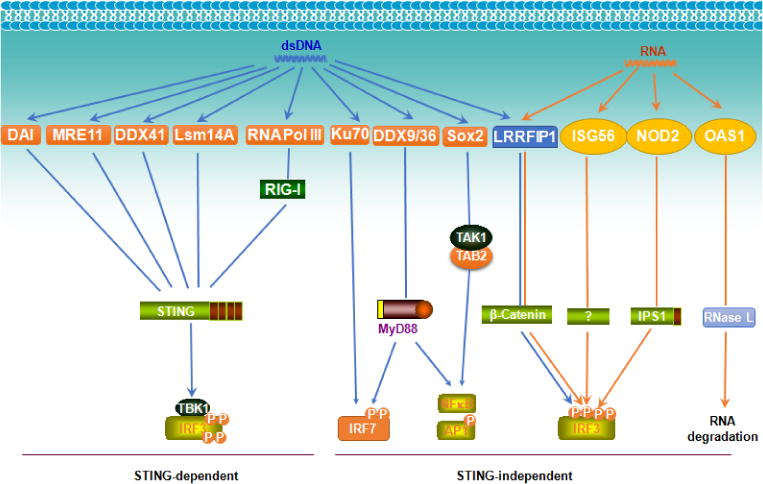
Cytosolic nucleic acid sensors triggering innate immune responses. An increasing pool of cytosolic “Toll-free” nucleic acid sensors have been identified in addition to cGAS-STING, RLRs, and the HIN200 family. They usually use STING to transmit their signal for the production of IFN-I via activating IRF3.

An increasing pool of individual cytosolic DNA and RNA sensors have been identified. Almost all these sensors trigger IFN-I production [13], except OASs that recognize dsRNA leading to RNA degradation by RNase L, and Sox2 that recognizes dsDNA triggering NFκB/AP1-mediated inflammation via activating the kinase complex TAK1-TAB2 (Figure 4) [8]. The adaptor STING converges different DNA sensing pathways for IRF3 activation leading to IFN-I production [53].

## 3. Evasion of “Toll-free” Innate Signaling Pathways

**Figure 5:**
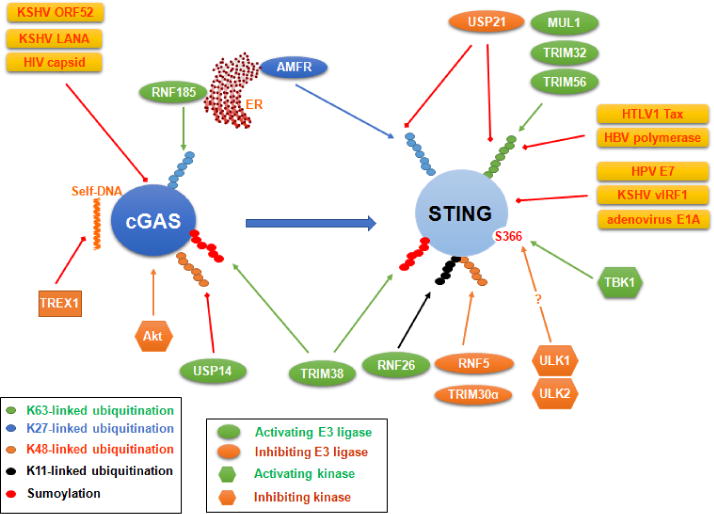
Regulation of cGAS and STING by cellular and viral factors. Phosphorylation and ubiquitination are two major posttranslational modifications that regulate cGAS and STING protein stability and activity. Many viral factors, with or without enzyme activity, may also regulate their activities through other mechanisms, including direct interaction and employment of cellular factors as mediators.

**Figure 6:**
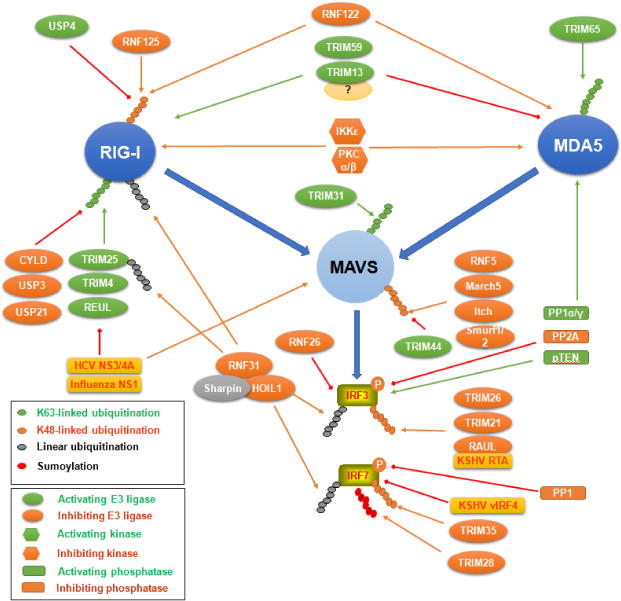
Regulation of RLR signaling components. The receptors RIG-I and MDA5, the adaptor MAVS, and the downstream IRF3/7 are shown as examples in the RLR signaling pathways for their regulation by phosphorylation and ubiquitination at the posttranslational level. Many viral factors may also regulate their activities through other mechanisms.

Innate sensing pathways are circumvented by numerous viral and cellular negative regulators (Figures 5 and 6). For example, Kaposi’s sarcoma-associated herpesvirus (KSHV)-encoded ORF52 and its homologs in other gammaherpesviruses (EBV, MHV68 and Rhesus monkey Rhadinovirus) inhibit cGAS activity to facilitate virus progeny [54,55]; KSHV-encoded vIRF1 negatively regulates STING-TBK1 interaction [56], and vIRF4 inhibits IRF7 transcriptional activity [57]. Some dsDNA and ssDNA viruses express proteins, including HPV E7 and human adenovirus E1A oncoproteins, with the Leu-x-Cys-x-Glu (LxCxE) motif that is known to block the Rb tumor suppressor [58]. The LxCxE motif mediates their interaction with STING to block IFN-I immune responses. Since these proteins are frequently used to immortalize cell lines [59], cGAS-STING pathway in these immortalized cell lines is compromised [50].

Nevertheless, those regulators with enzyme activities, such as DNA and RNA editing enzymes, kinases and phosphatases, ubiquitin E3 ligases and deubiquitinases, and histone modification enzymes, are especially interesting in that these regulators are potentially targeted with enzyme inhibitors for clinical interventions. In regard to evasion of HIV infection, these interesting enzymes include the RNA editing enzyme APOBEC3G, the ubiquitin E3 ligases TRIM5α and TRIM22, the exonucleases TREX1 and SAMHD1, and the GTPase Mx2, and they are all inducible by IFN-I. Tetherin (BST2), another IFN-inducible host restriction factor with broad antiviral activity (including HIV1), directly tethers virions for internalization, leading to endocytosis and subsequently degradation [60,61]. Apoptotic caspases have also been found to negatively regulate cGAS-STING pathway, although the precise mechanism remains unclear [24,62]. Of note, many PRRs themselves have enzymatic activity, including RLRs, cGAS, and DDXs, and some of their family members negatively regulate PRR signaling pathways; for example, DDX46 negatively regulates IFN-I responses by retaining antiviral mRNAs of TRAF3, TRAF6, and MAVS in the nucleus through eliminating their m6A modification [63].

Apart from protein regulators, miRNAs represent another interesting category of immune inhibitors with clinical potentials [64,65,66]. For example, miR-27 targets STING gene 3′UTR and represses its expression, resulting in impaired CCL22-mediated recruitment of Tregs in human papillomavirus (HPV)-positive tongue squamous cell carcinoma (TSCC) [67]. STING, in addition to MAVS and IRF3, is also targeted for downregulation by miR-576-3p [68]. Hypoxia induces Tet1-dependent expression of miR-25/93 that targets NCOA3, a lysine acetyltransferase that epigenetically induces cGAS expression in cooperation with AP1, resulting in suppression of cGAS expression in hypoxic breast cancers [69].

### 3.1. Evasion of nucleic acid sensing pathways by AGS exonucleases

The family of AGS exonucleases, including AGS1-5, are RNases except AGS1 (TREX1) that functions as a DNase. However, our recent in vitro biochemical evidence have shown that AGS1 can in fact also act as an RNase, which specifically degrades ssRNA [70]. Attraction is especially focused on AGS1 and AGS5 (SAMHD1) because of their roles in HIV1 infection. TREX1 is responsible for the degradation of self-DNA in the cytosol. In the absence of TREX1, viral infections including HIV1 infection cause accumulation of cytoplasmic DNA, which activates the cGAS-STING pathway leading to IFN-I production and eventually viral replication is inhibited. Deficiency of TREX1 also causes Aicardi-Goutieres syndrome, a childhood inflammatory disorder characterized by high endogenous levels of IFN-I resulting from activation of DNA sensors by excess endogenous DNA.

### 3.2. Evasion of RNA sensing pathways by RNA editing enzymes

The RNA editing enzymes in humans consist of the adenosine deaminases that act on RNA (ADARs, A-to-I) family and the cytidine deaminases (AID/APOBECs, C-to-U) family.

The double-stranded RNA (dsRNA) A-to-I adenosine deaminase acting on RNAs (ADARs) include ADAR1 (IFI4), ADAR2, and ADAR3. ADAR1 has two forms; the longer form p150 is IFN-inducible but the shorter form p110 is constitutively expressed, ADAR2 is constitutively expressed, and ADAR3 has no enzyme activity [71]. Bioinformatics analysis has indicated that only limited sites in human transcriptome are potentially edited by ADARs; however, it is estimated approximately 20% of the human miRNA precursors are subject to ADAR editing, and in IFN-mediated antiviral responses, specific editable sites are increased [71]. Also, retrotransposons, which represent nearly half of the mammalian genomes, undergo extensive A-to-I editing [72]. ADAR1 also competes with RIG-I and MDA5 for dsRNA binding independently of its RNA editing activity and therefore suppresses IFN response upon RNA virus infection [73], and also negatively regulates OAS1- and PKR-mediated immune responses. Due to its ability to act on both cellular and viral dsRNAs, ADAR1 has broad roles in repressing antiviral immunity and preventing autoimmune diseases [74]. However, for some other viruses, including HCV and influenza virus, A-to-I editing of viral RNAs promotes antiviral activity [74].

Furthermore, ADARs have been shown to regulate DNA virus latent infection such as the oncogenic KSHV and EBV that encode ADAR-targeted RNA transcripts [74]. Site-specific A-to-I RNA editing of KSHV oncogenic K15 transcript (encodes miR-K10 and kaposin A) abrogates its transforming activity and promotes KSHV replication [75]. The EBV pre-miR-BART6 transcript undergo A-to-I editing that negatively regulates miR-BART6 biological functions [76]. A-to-I editing of viral transcripts at multiple sites may alter ORFs and results in aberrant expression of viral proteins.

The AID/APOBEC C-to-U family, including eleven members, can selectively act on either RNA or ssDNA [77]. Among these members, AID and APOBEC3 (APOBEC3A-H), are of great interest due to their antiviral activity against HBV, HCV, HIV, HTLV1, HPV, and herpesvirus infection [77]. The ssDNA deaminase AID, which is primarily expressed in germinal center (GC) B cells and is the only enzyme known to induce oncogenic mutations in the human genome, is induced upon infection with HCV or *Helicobacter pylori*, and is also induced by EBV LMP1 and HTLV1 Tax via NFκB in their latency. APOBEC3 deaminases are only expressed in mammalians and inhibit retroviral infection by deaminating their DNA intermediates. For example, APOBEC3G defends viral infectivity factor 1 (VIF1)-deficient HIV infection; however, VIF1 causes APOBEC3G ubiquitination-dependent degradation. Similar to AID, APOBEC editing causes endogenous “off-target” DNA damage and genomic instability favoring cancer development, for example, in the setting of oncogenic HPV infection [77].

### 3.3. Evasion of type I IFN responses by protein kinases and phosphatases

Protein kinases usually activate PRR signaling by phosphorylating their components on specific sites. For example, the adaptors TRIF, MAVS and STING are phosphorylated at a serine site in a conserved motif pLxIS by TBK1 and IKKε for the recruitment of IRF3 [44], and phosphorylation of IRF3 and IRF7 by IKKs or IRAK1 is prerequisite for their activation leading to IFN-I production. STING Ser366 phosphorylation by TBK1 is believed to be required for its activation [44], although another report showed that phosphorylation of this site by autophagy-related kinases ULK1 (ATG1) and ULK2 diminishes STING activity [78]. However, site-specific phosphorylation of some components may cause their inactivation. For example, phosphorylation of human cGAS S305 (S291 in mouse) by Akt potently inhibits cGAS enzymatic activity [79]. Phosphorylation of IRF3 Ser97 inhibits its activity but the phosphatase pTEN releases this inhibition to promote IRF3-mediated IFN-I antiviral responses [80]. Phosphorylation of RIG-I by IKKε or PKCα/β negatively regulates its activity.

Recently, the roles of protein phosphatases in antiviral immune responses have emerged. PP1 and PP2A are positive regulators of Tat-dependent HIV1 transcription, and the ^35^QVCF^38^ motif of Tat directly interacts with PP1 [81]. PP1 also dephosphorylates the RNA sensors, MDA5 and RIG-I, and positively regulates their activity for IFN-I production in response to RNA virus infection [82,83]. Additionally, PP1 interacts with TRAF6, and promotes TRAF6-dependent innate immune responses [84].

In contrast, PP1 dephosphorylates Ebola virus VP30 and inhibits its transcription [85]. The measles virus V and the HBV X proteins can antagonize PP1 activity to facilitate their replication [83,86]. PP1 negatively regulates IRF3-mediated IFN-I production by inhibiting its phosphorylation at serines 396 and 385 [87], and together with GADD34, negatively regulates TLR-mediated immune response by inhibiting TAK1 serine 412 phosphorylation [88,89]. PP2A also deactivates IRF3 in cooperation with FBXO17 [90], and inhibits IFN-I signaling by inhibiting STAT1 phosphorylation and therefore contributes to HCV chronic infection [91]. More recently, we have identified PP1 as the first phosphatase that inactivates IRF7 by targeting its key phosphorylation sites and abrogates IRF7-mediated IFN-I responses [92]. Further verification of this important finding in mouse models in vivo is necessary.

### 3.4. Evasion of “Toll-free” innate pathways by ubiquitinases and deubiquitinases

Besides phosphorylation/dephosphorylation, ubiquitination/deubiquitination represent another major epigenetic modification that is committed to fine regulation of myriad cellular functions. It seems all components in each PRR signaling pathway, including the receptor, the adaptor, TRAFs, and downstream IRFs, are regulated by ubiquitination (Figures 5 and 6) [93,94,95]. The involved cellular E3 ligases mainly include the TRAF, TRIM, and RNF families, and the cellular deubiquitinases mainly include the OTU and USP families [96,97]. For example, the deubiquitinases Gumby (OTULIN), OTUD7b, and TNFAIP3 (A20) in the OUT family were shown to negatively regulate NFκB activation through different mechanisms in different biological contexts [98–104].

The ER-anchored RNF185 triggers K27-linked ubiquitination of cGAS promoting its activation [105]; AMFR-mediated K27-linked ubiquitination and TRIM32, TRM56, or MUL1-mediated K63-linked ubiquitination of STING are required for its activation [45,106,107,108], but HTLV1 Tax and HBV polymerase negatively regulate STING K63-linked ubiquitination, and the cellular E3 ligases RNF5 and TRIM30α triggers STING K48-linked ubiquitination and degradation to alleviate cytosolic DNA sensing pathways [109–112]. RNF26 protects STING from RNF5-mediated degradation by targeting the same site (K150) for K11-linked ubiquitination, whereas promotes autophagy-mediated degradation of IRF3 [113]. USP21, a deubiquitinase, is recruited to STING after HSV1-stimulated phosphorylation via p38 and negatively regulates STING activity by hydrolyzing K27- and K63-linked ubiquitin chains of STING [114].

TRIM65 triggers K63-linked ubiquitination of MDA5 [115], and TRIM31 triggers K63-linked ubiquitination of downstream adaptor MAVS [116], both promoting RIG-I signaling pathway; whereas the membrane-anchored TRIM13 and TRIM59 negatively regulate MDA5-mediated IFN-I responses but promotes RIG-I activity through unclear mechanism that may involve additional cofactors [117]. TRIM4, TRIM25/RNF147, and Reul/RNF135/RIPLET E3 ligases promotes RIG-I K63-linked ubiquitination and activation [118–122]. In contrast, RNF122 and RNF125 target RIG-I for ubiquitination-mediated degradation [123,124].

TRIM21 targets IRF3 and DDX41, and TRIM26 and SENP2 target IRF3, for degradation, and TRIM21 also negatively regulates FADD-mediated antiviral immunity. TRIM35 targets IRF7 for degradation. We have shown that TRAF6 E3 ligase promotes IRF7 K63-linked ubiquitination that is required for EBV LMP1 activation of IRF7 [125]; however, A20, a member with both E3 ligase and deubiquitinase activities in the OTU family, inhibits LMP1-stimulated IRF7 activity by acting as a deubiquitinase [126].

In recent years, LUBAC-mediated linear polyubiquitination is coming into focus due to its emerging role in activation of NFκB in response to diverse signaling stimuli [127–132], including apoptotic and immune stimuli such as TNFα [133,134], IL1β [135], genotoxic stress [136], CD40 [137], Toll-like receptors (TLRs) [138], NOD2 [98], and NLRP3 [139]. LUBAC is a ternary ubiquitin ligase complex composed of HOIP (RNF31), HOIL1L (RNF54), and SHARPIN, and is constitutively formed under normal physiological conditions [133,134,140]. RNF31 is likely the central component of this complex [141]. LUBAC components are abundantly expressed in thymus and spleen, implying its potential role in lymphocytes [130]. Recent reports clearly show that LUBAC specifically activates the canonical NFκB pathway, but not the JNK pathway, by conjugating linear polyubiquitin chains onto NEMO and RIP1 [130,142]. However, LUBAC negatively regulates RIG-I-mediated innate immune responses by targeting RIG-I, TRIM25 and IRF3 for degradation [143,144], and by disrupting the TRAF3-MAVS complex [145]. We have recently shown that LUBAC-mediated linear ubiquitination is required for LMP1 activation of NFκB, but inhibits LMP1-stimulated IRF7 activity [146].

Compared with host cellular factors, virus-encoded factors are of greater interest in that they are virus-specific and easier to manipulate without affecting bypass cellular functions. Herpesvirus-encoded E3 ligases have been found to play crucial roles in evading innate immune responses for facilitating their infection and establishment of latent infection. For example, HSV1 ICP0 and HCMV pUL83 ubiquitin E3 ligases target IFI16 for proteasome-dependent degradation [147,148]. KSHV IE lytic transactivator RTA/ORF50 functions as a ubiquitin E3 ligase that targets IRF7 for degradation [149]. Herpesviruses also encode deubiquitinases, many of which have also been shown to evade innate immune responses. For example, EBV-encoded BPLF1 functions as a deubiquitinase that suppresses NFκB activation downstream of TLR signaling [150].

Ubiquitination-like modifications, especially sumoylation, have been shown to exert similar functions to ubiquitination in antiviral regulation. For example, TRIM38 stabilizes cGAS and STING by promoting their sumoylation [151], and TRIM28 is a SUMO E3 ligase that promotes IRF7 sumoylation and negatively regulates its activity [152]. These intriguing findings have advanced our current understanding of the novel functions of ubiquitination-like posttranslational modifications in signal transduction pathways [153,154].

## 4. Closing Remarks

The increasing pool of cytosolic nucleic acid receptors have greatly advanced the study of IFN-I-mediated innate immune mechanisms, and may also shed light on type III IFNs-mediated responses that involve similar induction mechanisms to IFN-I. It is plausible that better understanding of innate immune pathways will benefit clinical applications for both antiviral infections and inflammation-related diseases including cancers. Recent research brings cGAS-STING pathway to the focus for potential treatments of cancers in that this pathway plays crucial roles in antitumor immunity and is aberrantly regulated in many cancers [28]. Future efforts may concentrate on the identification of viral and cellular regulators of these signaling pathways so as to design appropriate strategies such as cancer vaccines to evoke or interfere with their functions in different related disease settings, including cancers and autoimmune disorders.
